# Synthesis and characterization of biodegradable palm palmitic acid based bioplastic

**DOI:** 10.3906/kim-2011-31

**Published:** 2021-06-30

**Authors:** Abd Al-Wali JAPIR, Nadia SALIH, Jumat SALIMON

**Affiliations:** 1 Department of Chemistry, Faculty of Education, Thamar University, Thamar Yemen; 2 Department of Chemical Sciences, Faculty of Science and Technology, University Kebangsaan Malaysia, Bangi, Selangor Malaysia

**Keywords:** Polyesters, methyl palmitate, dimethyl 2-tetradecylmalonate

## Abstract

This study involves the quantitative analysis of high free fatty acid crude palm oil, the separation of palmitic acid and synthesis of palm palmitic acid-based bioplastic. Synthesis of dimethyl 2-tetradecylmalonate (DMTDM) using methyl palmitate (MP) with sodium hydride (NaH) in the presence of reactive solvent of dimethyl carbonate (DMC) was carried out. The reaction conditions comprise at a mole ratio of MP: DMC: NaH: dimethylformamide (DMF) (0.1:2:0.25:1) at 60 °C for 14 h with 88.3 ± 1.4% yield. FTIR spectra of DMTDM showed the ester carbonyl group at 1740 cm^–1^. The polymerization of DMTDM with 1,6-hexandiol or 1,12-dodecandiol was carried out using titanium (IV) isopropoxide Ti(OiPr)_4_ as the catalyst and reaction time of 24 h. The results showed that the poly(dodecyl 2-tetradecylmalonte) (PDTDM) exhibited good thermal properties compared to poly(hexyl 2-tetradecylmalonte) (PHTDM). The increase of the chain length of diol in PDTDM improved the thermal properties of polyester with glass transition, T_g_ of 13 ºC and melting point of 51 ºC with a molecular weight of 12508 Da and polydispersity index (PDI) of 1.4. In general, the synthetic polyesters can be used as internalplasticizer in bio-based industry.

## 1. Introduction

Palmitic acid is a saturated long-chain fatty acid with a 16-carbon backbone. It is naturally found in some animal products such as meat and dairy, as well as in palm, palm kernel, and coconut oils. Because these two oils are frequently used in processed foods, you might be getting palmitic acid in your diet without even realizing it. One of the main uses of palmitic acid is in soaps because of its ability to help keep skin smooth. Palmitic acid is found in beeswax, which is a popular ingredient in personal care products. In cosmetics, palmitic acid is used in skin make-up to hide blemishes. It is also used in certain surfactants as a cleaning agent. The U.S. Food and Drug Administration (FDA) has listed palmitic acid as a generally recognized as safe (GRAS) substance, which classifies palmitic acid as safe as an additive to food and in the manufacture of food components [1].

The rapid industrial development witnessed over the years is largely dependent on the use of fossil materials, mainly coal and crude oil. This explains the extensive use of crude oil and coal in various areas of life, such as plastics to the relatively low prices. Up to the present time, the crude oil and its derivatives are the most important raw materials for the oleochemical industry. Nevertheless, crude oil as a fossil source is depleting raw material. A few researches have introduced the use of animal fat and plant oils as feedstock for the synthesis of biodegradable polymers such as bioplastic in place of fossil source. Moreover, the polymer is a biodegradable, environmentally friendly, and substitute materials for polymers derived from petroleum resources [2].

According to the European Bioplastics organization, bioplastics can be defined as biodegradable plastics or plastics produced from renewable resources (biobased). Presently, the awareness in the packaging industry regarding the use of bioplastics is low, as their properties are unlike from the crude oil-based plastics such as polyethylene and polyethylene terephthalate [3]. Generally, plastic materials play a significant role in daily life. Their physical and mechanical features make them quite suitable for usage. However, nondegradable plastics accumulate in the environment at the rate of 25 million tons per year [4]. Hence, the researchers have to find an alternative solution via producing biodegradable polymers. 

Bioplastics production is normally synthesized via biological pathway such producing of polyhydroxyalkanoates (PHA) using microbial processing of biobased materials such as fatty acids via different types of bacteria [5,6]. Meanwhile, these methods are widely available and quite expensive. However, the continued reliance on edible oils as feedstocks for bioplastics production has threatened the supply of edible oil to the food industry and increased some environmental problems such as severe destruction of vital soil resources, deforestation, and usage of much of the available arable land. Furthermore, in the last ten years, the prices of plant oils have gradually increased which will affect the economic feasibility of bioplastic industry. Due to these factors, it is crucial to find other alternative oil feedstock to substitute edible oil in the production of bioplastics [7]. Then, the main aim of this study is to produce bioplastics from cheap resource and nonedible such as high free fatty acid crude palm oil (HFFA-CPO) using chemical pathway. Up to date, there are very few reports on production of polyester bioplastic-based palm palmitic acid (PA), possibly because the conversion of saturated fatty acids (SFAs) into monomer polyesters is a challenging task. 

In this study, saturated palmitic acid was used as a starting material for the production of a new kind of bioplastic with intrinsic properties different from those of conventional bioplastic products. Palmitic acid was defunctionalized by making it involves two functional groups to ensure the easy synthesis of polyester. This work is considered relatively novel because there are only very few reports on the synthesis of bioplastics based on palm palmitic acid via a chemical pathway.

## 2. Materials and methods

### 2.1. Oil sample

The HFFA-CPO used for this study was provided by Sime Darby Company, Carey Island, Selangor, Malaysia. The samples were stored at 4 ºC.

### 2.2. Chemicals

The chemicals and solvents used in the current study comprise hydrochloric acid (37%), toluene (99%), *n* -hexane (99%), anhydrous sodium sulphate (99%), dimethyl carbonate (99%), sodium hydride (60 wt% suspension in mineral oil), *N* , *N* -dimethylformamide (99.5%), 1, 12-dodecandiol (99%), 1,6-hexanediol (98%), titanium (IV) isopropoxide (98%) (Ti(OiPr)_4_), and tetrahydrofuran (99.5%). These chemicals and solvents were either analytical grade or high performance liquid chromatography (HPLC). Thus, they required no further purification.

### 2.3. Instrumentation

Calorimetric measurements of palm palmitic acid based polyesters were conducted using a differential scanning calorimetry (DSC) thermal analysis system in the range of –50 to 500 ^º^C at a heating rate of 20 ^º^C/min. The glass transition temperature (T_g_) and crystalline melting point (T_m_) were determined from the DSC thermogram of the second scan. The nuclear magnetic resonance analysis (NMR) for proton ^1^H and ^13^C (NMR) was carried out according to Gunstone et al. (2007) [8]. The spectra were recorded on a Bruker AV400111 (Bruker, Billerica, MA, USA) 400 MHz at 30 ^º^C. Tetramethylsilane (TMS) was used as an internal chemical shift reference. About 25 mg of sample was dissolved in 1 mL of deuterated chloroform (CDCl_3_) in all experiments. Fourier transforms infrared spectroscopy (FTIR) was carried out according to Sherazi et al. (2009) [9]. FTIR spectra of the products were recorded (Perkin Elmer Spectrum GX spectrophotometer, PerkinElmer, Waltham, MA USA) in the range of 400–4000 cm^–1^. A very thin film of sample was covered on NaCl cells (25 mm id × 4 mm thickness) was used for the analysis. GC-FID (gas chromatography-flame ionization detector) analyses were performed using gas chromatography (Model 5890 Series II GC, Hewlett Packard, Houston, TX, USA) software equipped with flame ionization detector (FID) and a BPX-70 fused silica capillary column (30 m, 0.25 mm id, 0.25 μm film thickness). GC-MS (gas chromatography-mass spectrometer) analysis was performed with Agilent 7890A gas chromatography (GC) equipped with the mass spectrometer system (Agilent 5975C inert MSD with Triple-Axis Detector, Agilent Technologies, Santa Clara, CA, USA). A fused silica capillary column was used: DB-5 (30 m × 0.25 mm id, film thickness 0.25 μm).

### 2.4. Polyesters molecular weight (MW) determination

The molecular weight of the synthesized polyesters comprising a number of average molecular mass (^–^ *M* *_n_* ), weight of average molecular mass (^–^M_w_), and polydispersity index (PDI) were determined using 1515 isocratic HPLC pump and Waters 2414 gel permeation chromatography (GPC) (Waters Corporation, Milford, MA, USA), with a differential refractive index meter. The mobile phase was THF and the column was calibrated with polystyrene standards (Shodex Standards, SL-105, Showa Denko, Tokyo, Japan). About 0.02–0.05 g of polyester was dissolved in 10 mL THF. The solution was filtered using a 0.45 μm syringe filter before bringing injected into the GPC system. A 50 μL of polymer solution was injected into the GPC system, the temperature of the columns was kept constant at 40 ºC in a column oven and the flow rate was 1 mL/min. The data was processed by using Breeze software.

### 2.5. Synthesis of palmitic acid based bioplastic

#### 2.5.1. Methyl palmitate synthesis

Approximately 0.008 mole (2 g) of PAwas added to a small (50 mL) two-neck round-bottom flask. Afterward, 0.2 mole (8.7 mL) methanol was mixed with 0.01 mole (0.3 mL) of HCl 37% and followed by the addition of 1.4 × 10^–2^ mole (1.5 mL) toluene. The mixture was subsequently refluxed at 65 ºC for 1.5 h. The mixture was then transferred into a separation funnel, where 10 mL of distilled water and 15 mL of hexane were added. After that, the mixture was left to stand until two-layer separation was achieved. The organic upper layer containing methyl palmitate (MP) was separated and dried overnight using anhydrous sodium sulphate (Na_2_SO_4_). The hexane was subsequently recovered under low pressure using a vacuum rotary evaporator at 35 ºC.

#### 2.5.2. Synthesis of malonate derivative (monomer)

Synthesis of malonate derivative was carried out in line with Kolb and Meier (2012) [10]. About 27.04 g of the methyl palmitate (0.1 mol) was added to 170 mL dimethyl carbonate (DMC) (2 mol), 10 g sodium hydride (NaH) (0.25 mol) and 7.7 mL *N* , *N* -Dimethylformamide (DMF) (1 mol). The mixture was placed in a 500 mL round bottom two-necked flask and refluxed at 60 ºC for 14 h. Diluted hydrochloric acid (10%) (200 mL) was added slowly to the suspension in order to halt the reaction. The malonate derivative (organic phase) was separated and concentrated under low pressure. High vacuum distillation was used for isolation and purification of malonate derivative, the yield of dimethyl 2-tetradecylmalonate was measured.

#### 2.5.3. Synthesis of polyester

About 3.28 g of dimethyl 2-tetradecylmalonate (10 mmol) was placed in a 100 mL round bottom three-necked flask and was mixed with 10 mmol of diol (1.182 g of 1,6-hexanediol or 2.023 g of 1, 12-dodecanediol) and 30 μL of Ti(OiPr)_4_ (1 mol%). Then 4.0 mL of tetrahydrofuran (THF) was subsequently added to the mixture in order tohomogenize the contents, which was then heated to 120 ºC for 1 h under a stream of nitrogen where the THF was evaporated after 1–2 min. Afterward, the reaction was completed under high vacuum (10^–2 ^mbar) at 120 ºC for 23 h. The polyester was then dissolved in THF and precipitated from methanol at ambient temperature.

## 3. Results and discussion

### 3.1. Synthesis of methyl palmitate (MP)

**Figure 1:**
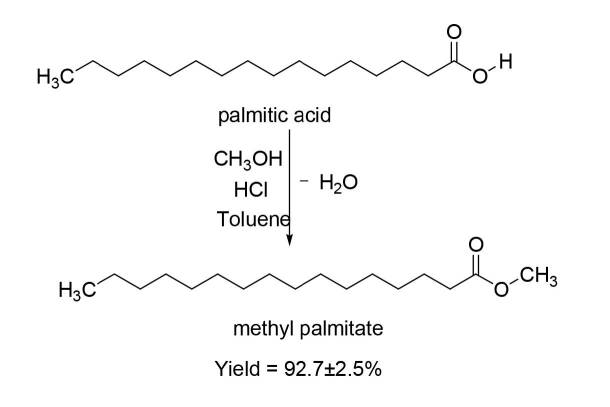
Synthesis of methyl palmitate.

MP was synthesized successfully using acid catalyzed esterification of palmitic acid with a yield of 92.7 ± 2.5%, as shown in Figure 1.

#### 3.1.1. FTIR analysis of MP

**Figure 2:**
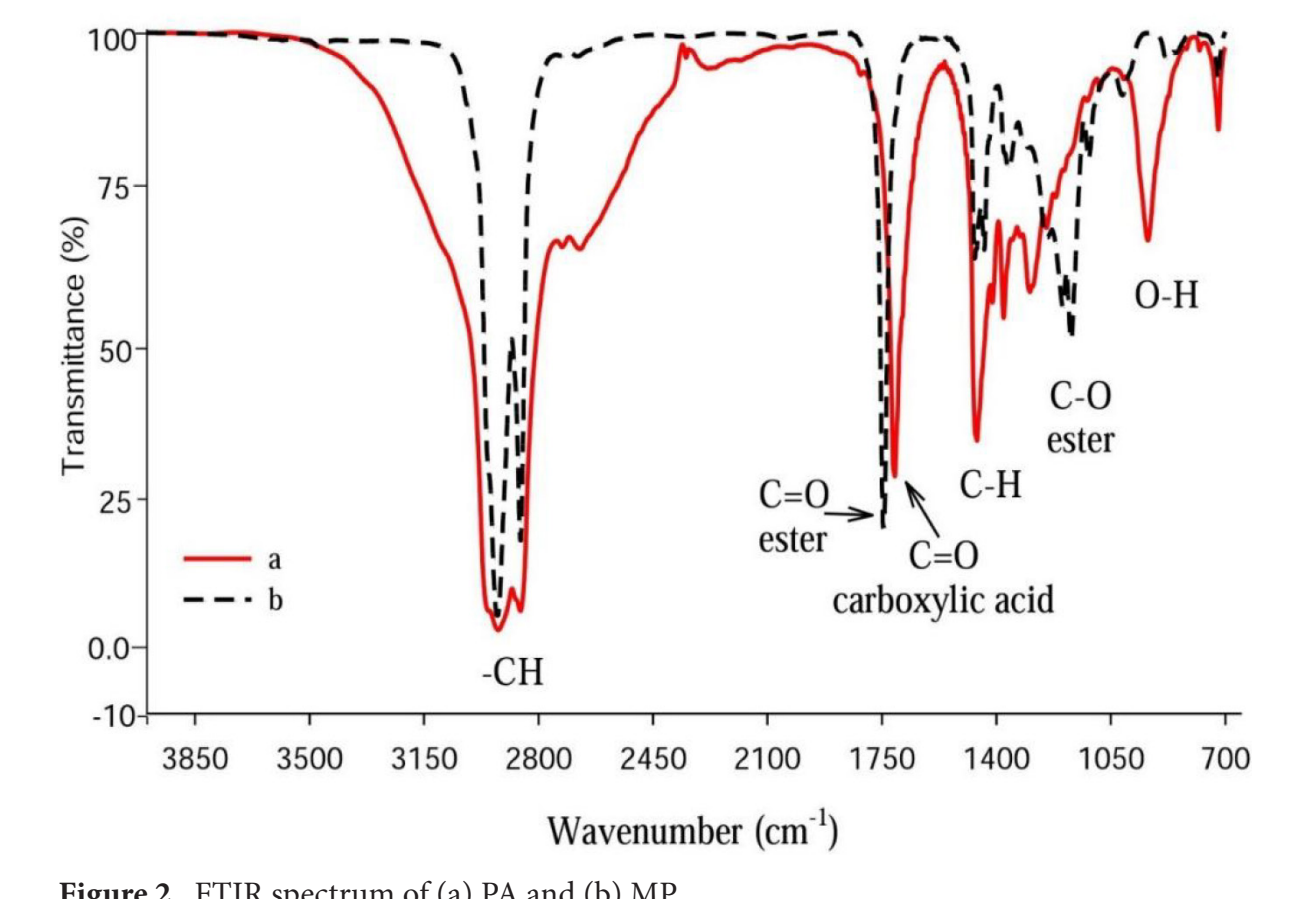
FTIR spectrum of (a) PA and (b) MP.

To confirm the presence of a carbonyl ester group of MP, which is the product of PA transesterification, FTIR analysis was carried out (Figure 2). Distinct absorption bands of high intensity at 1710 cm^–1 ^and1745 cm^–1^ for PA and MP, respectively, are assigned to functional groups of carbonyl C=O carboxylic acid and esterified carbonyl C=O. In addition, PA spectrum shows a typical broad band at around 3100 cm^–1^ referring to stretching of H-bonded, while this band is absent in MP. This difference ascertains the formation of MP. The peaks at 2922–2856 cm^–1^ indicate the CH_2_ and CH_3_ stretching vibration of PA and MP. The absorption band at 722 cm^–1 ^indicates (C-H) group vibration for PA and MP. A Sharp absorption band of medium intensity at 937 cm^–1^is assigned to out-of-plane deformation and hydroxy –OH carboxylic acid of PA but was disappeared in MP. 

#### 3.1.2. NMR analysis of MP

**Figure 3:**
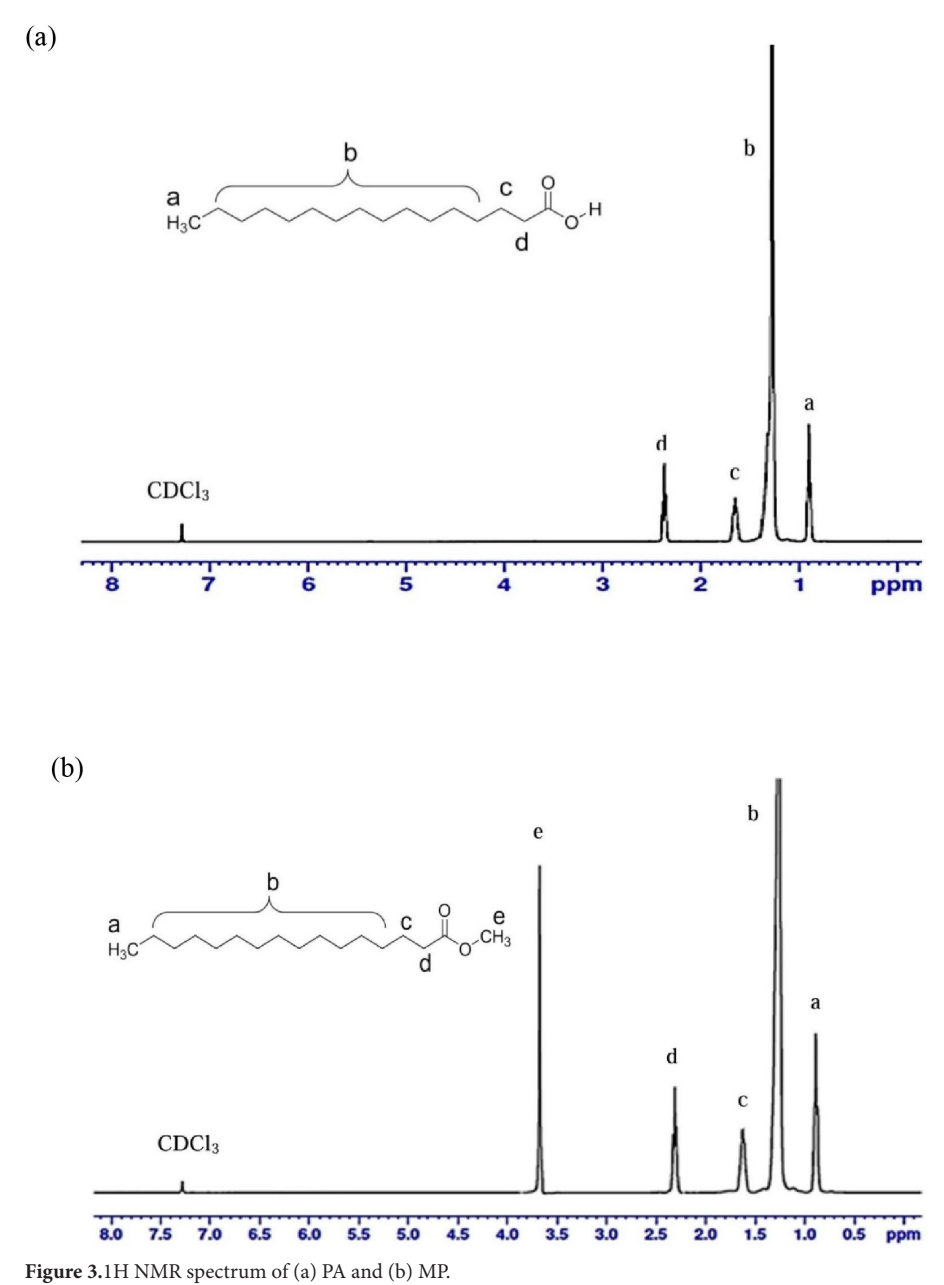
1H NMR spectrum of (a) PA and (b) MP.

The ^1^H NMR spectroscopy displays the major signal assignments in PA and MP (Figure 3). The signal at 0.88–0.92 ppm is assigned to the terminal methylene group (-CH_3_) of PA, which also appeared in MP spectrum at 0.87–0.90 ppm close to the terminal methyl from C-4 to C-15 (–CH_2_) at 1.27–1.32 ppm of PA, 1.26 ppm of MP. However, β- and α-methyl carbon in the acidic group were deshielded and absorb downfield at about 1.61–1.67 ppm, 2.35–2.39 ppm for PA and 1.61–1.63 ppm, 2.29–2.33 ppm for MP, respectively. The signal at 3.68 ppm is attributed to methyl ester (-COO-CH_3_) which appears in MP. The above spectral features of PA and MP are in conformity with those observed in the studies by Maria and Antonio [11] and Liu et al. (2015) [12], respectively. 

**Figure 4:**
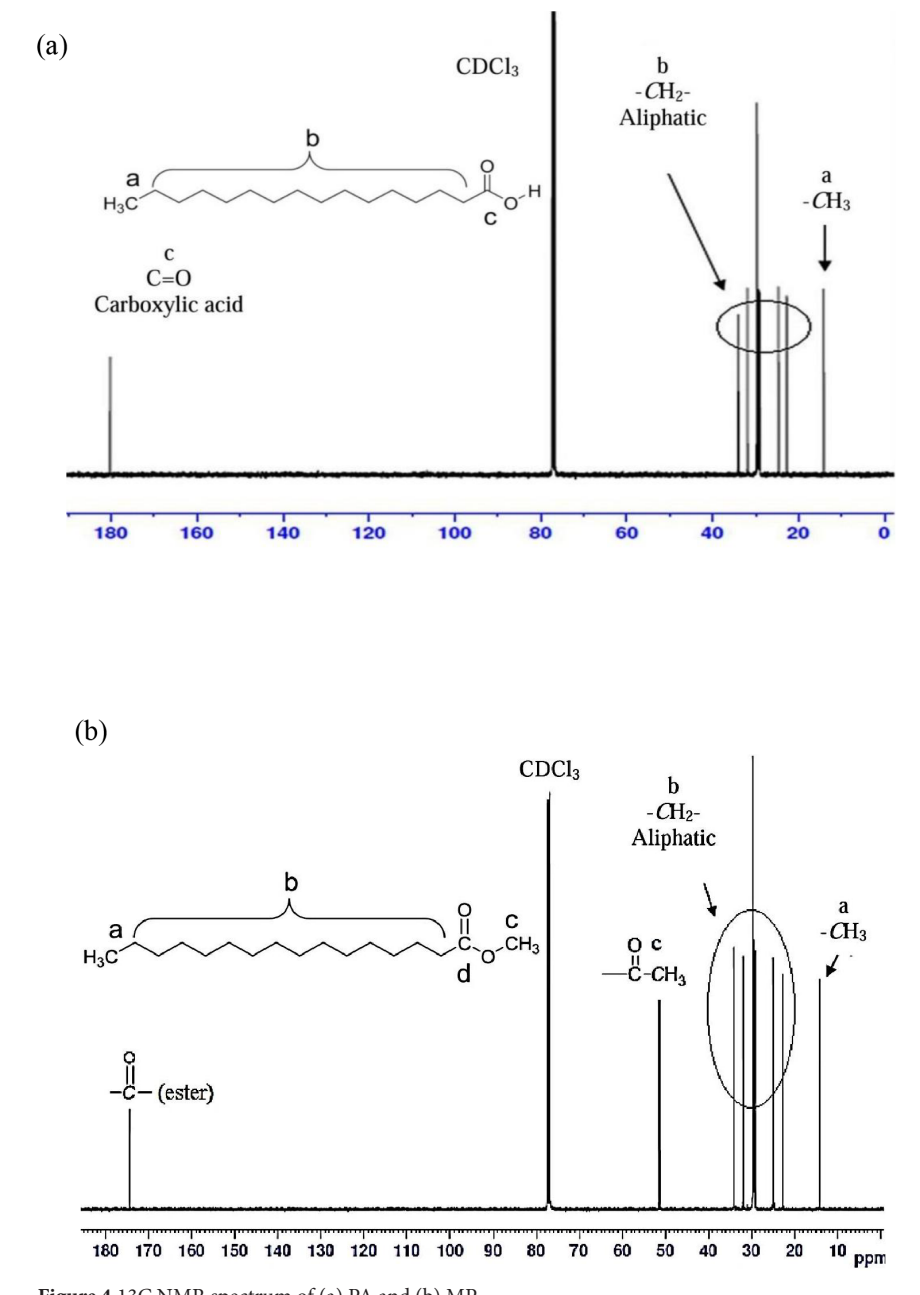
13C NMR spectrum of (a) PA and (b) MP.

The ^13^C spectroscopy presents the major signal assignment of the PA. The signal at 180.27 ppm refers to carbon atom of the carbonyl group (carboxylic acid). The signals at 14.16–34.09 ppm refer to aliphatic carbon. Figure 4 indicates the ^13^C NMR spectrum of PA and MP. The ^13^C spectroscopy presents the major signal assignment of the MP. The signal at 174.38 ppm refers to carbon atom of the carbonyl group (ester). The signals at 14.13–34.12 ppm denote aliphatic carbon. However, the carbon atom that attached to ester group was deshielded and absorb downfield at about 51.45 ppm.

### 3.2. Synthesis of malonate derivative (dimethyl 2-tetradecylmalonate) (DMTDM)

**Figure 5:**
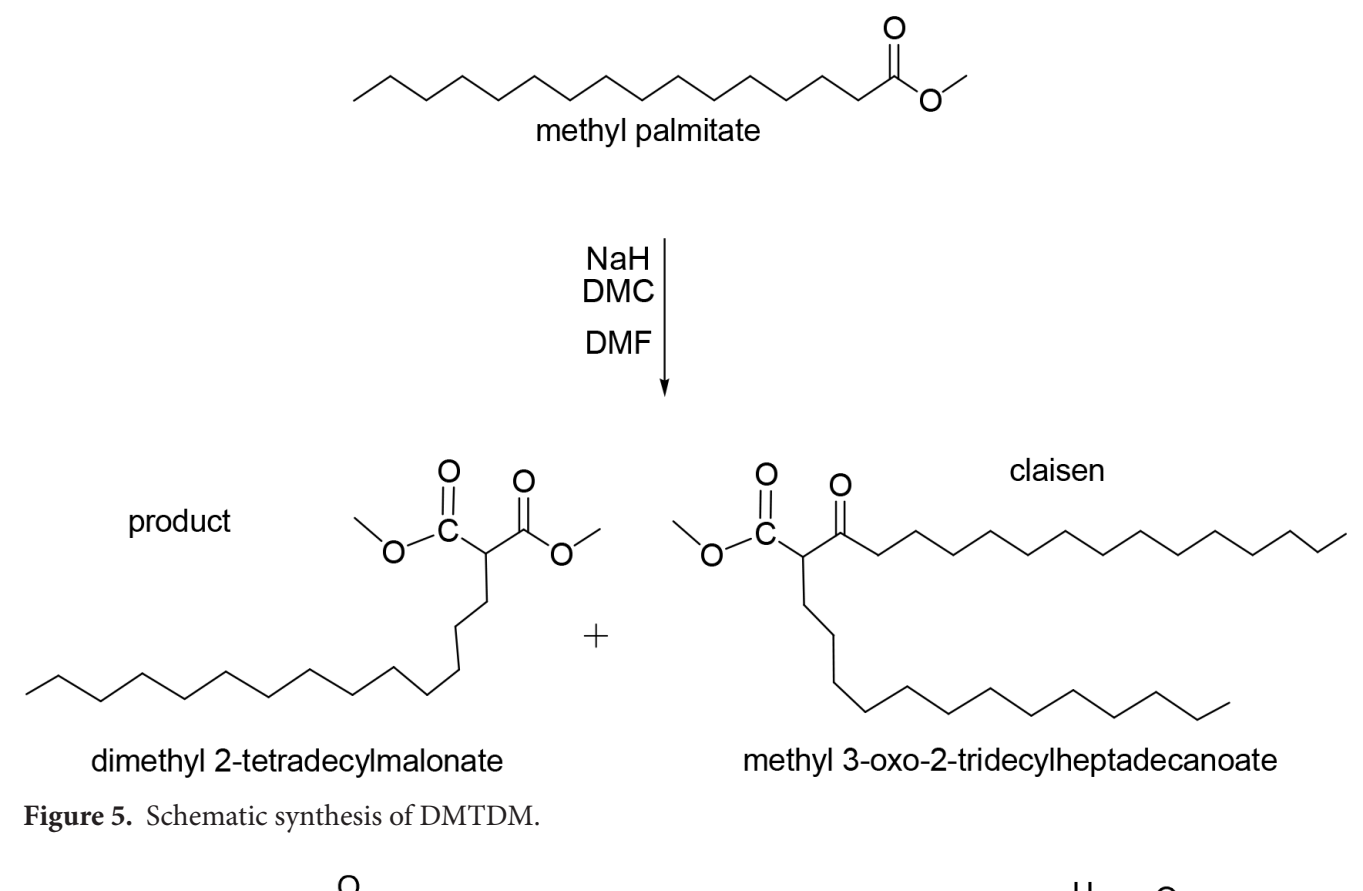
Schematic synthesis of DMTDM.

The functionalization of methyl palmitate was successfully done deprotonating the α-position of the ester with a strong base such as sodium hydride (NaH) in a reactive solvent, specifically dimethyl carbonate (DMC). When methyl palmitate enolate is formed first, it either reacts with another methyl palmitate to undergo a Claisen condensation reaction [13] or in a nucleophilic manner with DMC to form DMTDM (Figure 5). Tundo et. al. (2018) [14] reported that DMC can be an attractive eco-friendly alternative to toxic organic solvents and is considered as a “green solvent” due to it is relatively safe nature when compared to other organic solvents and can be prepared from methanol and carbon monoxide or dioxide. 

**Figure 6:**
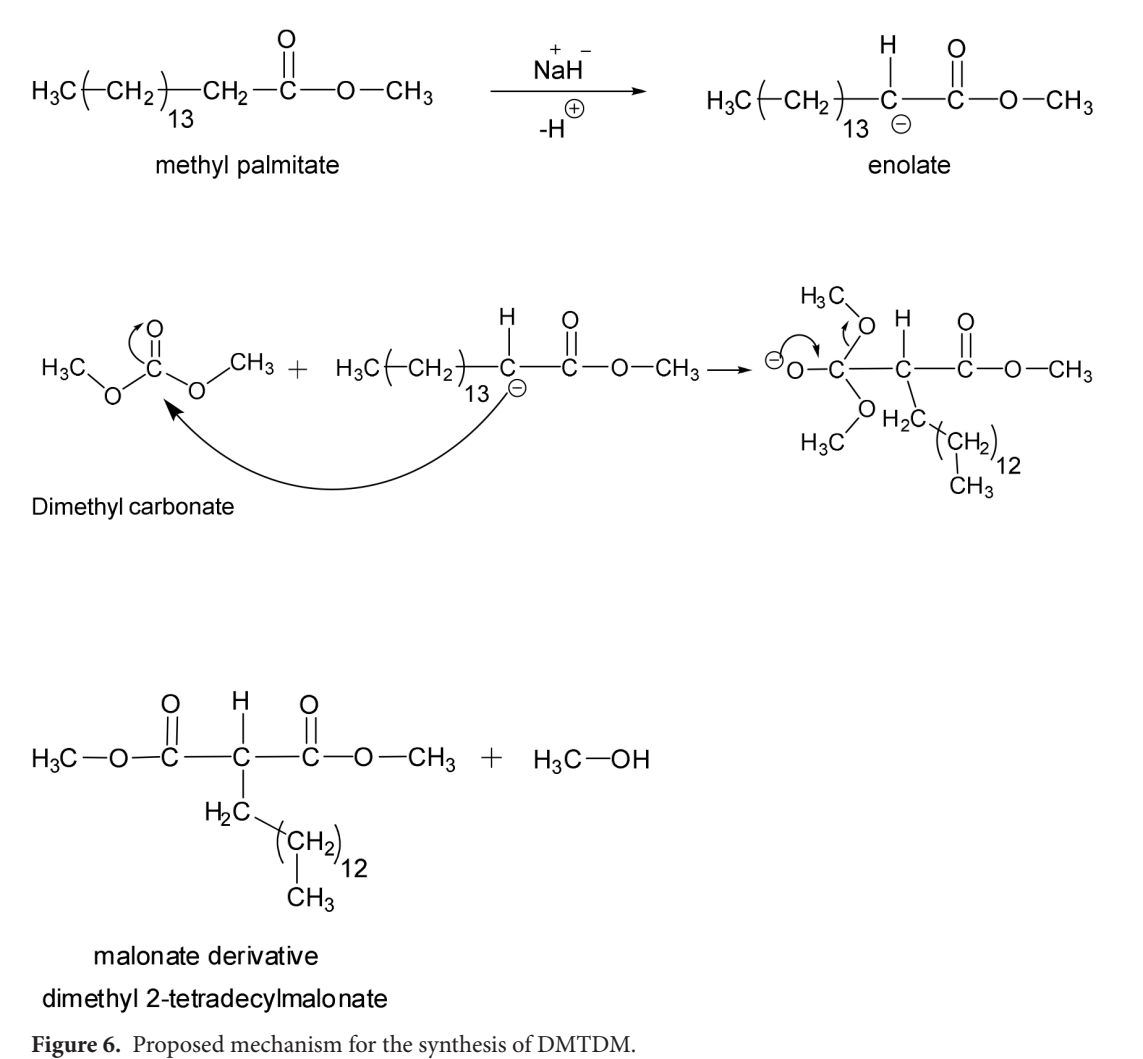
Proposed mechanism for the synthesis of DMTDM.

Figure 6 illustrates the mechanism reaction was proposed according to Claisen condensation. The first step includes the formation of ester enolate ion by reaction of a strong base (NaH) with methyl palmitate. The second step includes the addition of the enolate ion to dimethyl carbonate (DMC) to give tetrahedral intermediate. The third step includes elimination of alkoxide leaving group and formation of malonate derivative. However, in Claisen condensation the final product is β-ketone ester or a β-diketone. Therefore, in the reaction of methyl palmitate with DMC, the final product is malonate derivative due to increasing the mole ratio of DMC to suppressing Claisen condensation.

To select a good product and achieve high conversation yield, it is important to optimize the reaction conditions. The reaction conditions were found to be 20 eq. DMC, 2.5 eq. NaH, and 1 eq. DMF was added as an additive at 60 ºC for 14 h, thus suppressing the Claisen condensation as a side-reaction according to Kolb and Meier (2012) [9]. It has been found that the addition of 1 eq. DMF accelerates the reaction by acting as a good solvation for intermediates and reactants, thus increasing reactivity [15, 16]. Dimethyl carbonate was utilized in a large excess of 20 eq. to suppress Claisen condensation side-product. The yields were lower because of the large dilution. Therefore, the reaction time was prolonging to 14 h to ensure obtaining of relatively high yield and good conversion as well as low percentage of Claisen condensation side-product. A disadvantage of this reaction in terms of safety and sustainability is that a large excess of NaH was required because of the malonate derivative is more acidic than the starting ester-methyl palmitate and is thus favorably deprotonated. Therefore, base loadings less than 2.0 eq. led to very low conversions as reported by Kolb and Meier (2012) [9].

#### 3.2.1. GC-FID and GC-MS analysis of DMTDM

**Figure 7:**
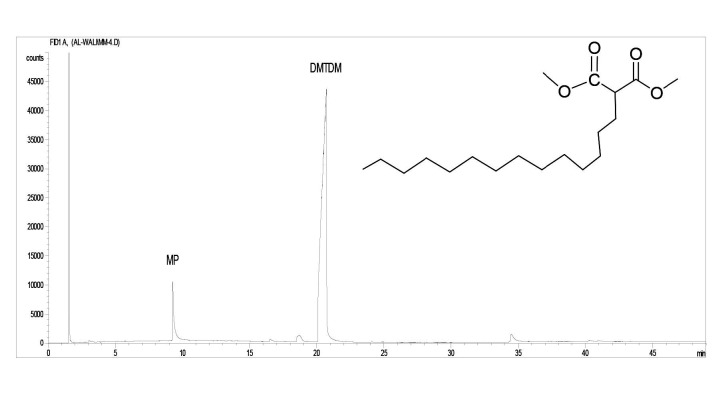
GC-FID chromatogram of DMTDM.

**Figure 8:**
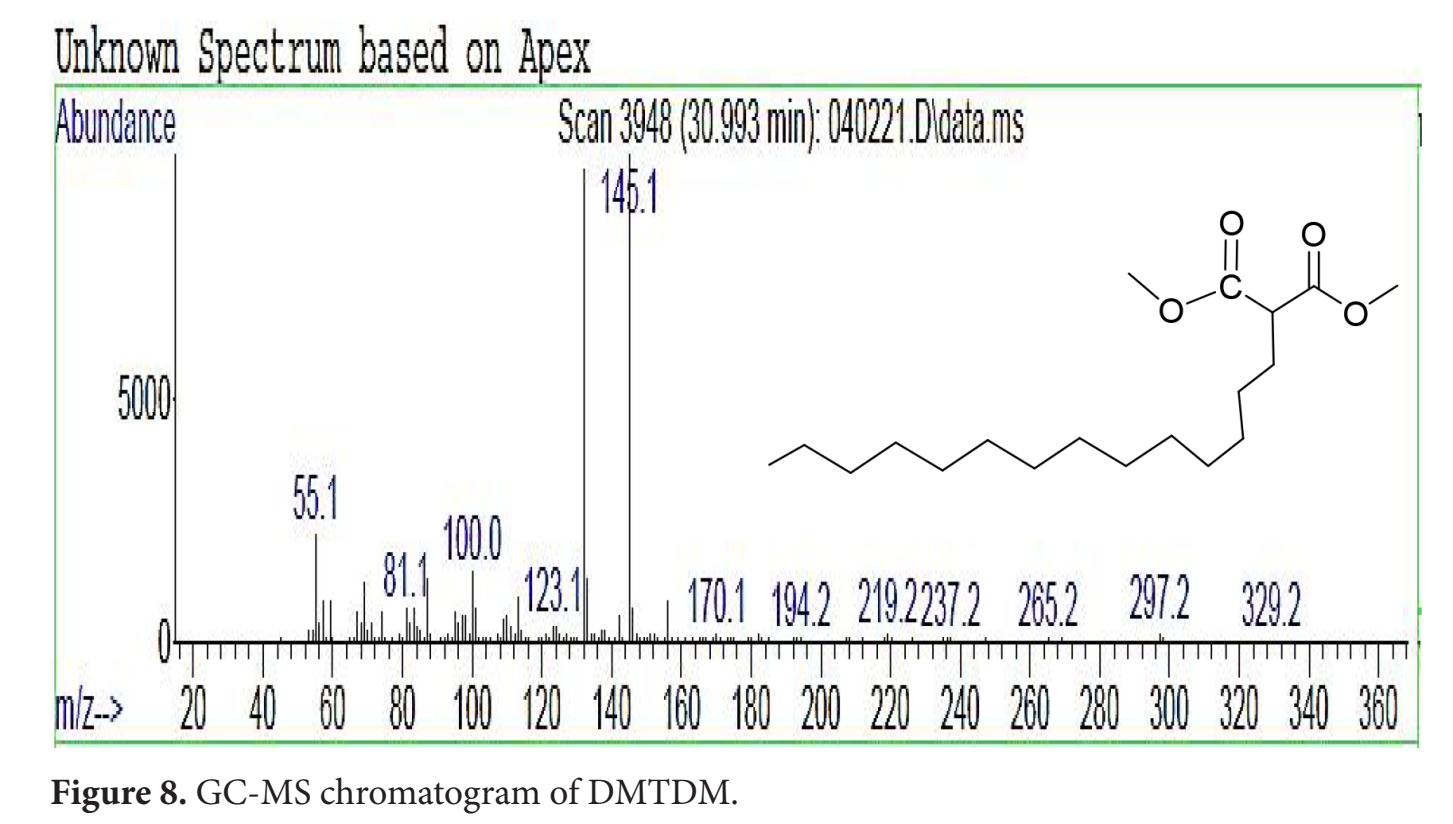
GC-MS chromatogram of DMTDM.

The final product of DMTDM was a white wax with a yield of 88.3 ± 1.4%. DMTDM was subjected to copolymerization without any further purification due to its high purity with 94.7% GC and *m/z* =329.2 ([M] calc. 328.3). Figures 7 and 8 show the GC-FID and GC-MS chromatogram of DMTDM, respectively.

#### 3.2.2. FTIR analysis of DMTDM 

**Figure 9:**
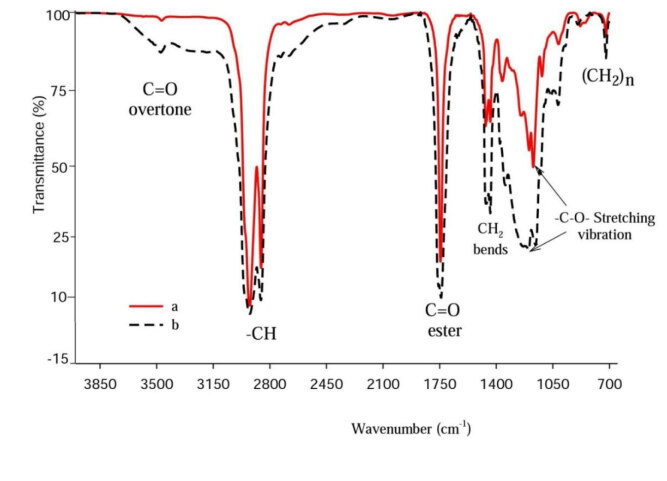
FTIR spectrum of (a) MP and (b) DMTDM.

To prove the formation of dimethyl 2-tetradecylmalonate, which is the main product of the reaction of MP with NaH in the presence of DMC, the sample was analyzed with FTIR. The FTIR spectrum of MP and DMTDM is displayed in Figure 9. The sharp absorption bands of high intensity at 1745 cm^–1^ and 1740 cm^–1 ^for both MP and DMTDM are assigned to functional groups of esterified carbonyl C=O, respectively. The peaks at 2925–2855 cm^–1^ indicate the CH_2_ and CH_3_ stretching vibration of MP and DMTDM. The absorption bands at 722 cm^–1^ are related to (C-H) group vibration for MP and DMTDM. 

#### 3.2.3. NMR analysis of DMTDM 

**Figure 10:**
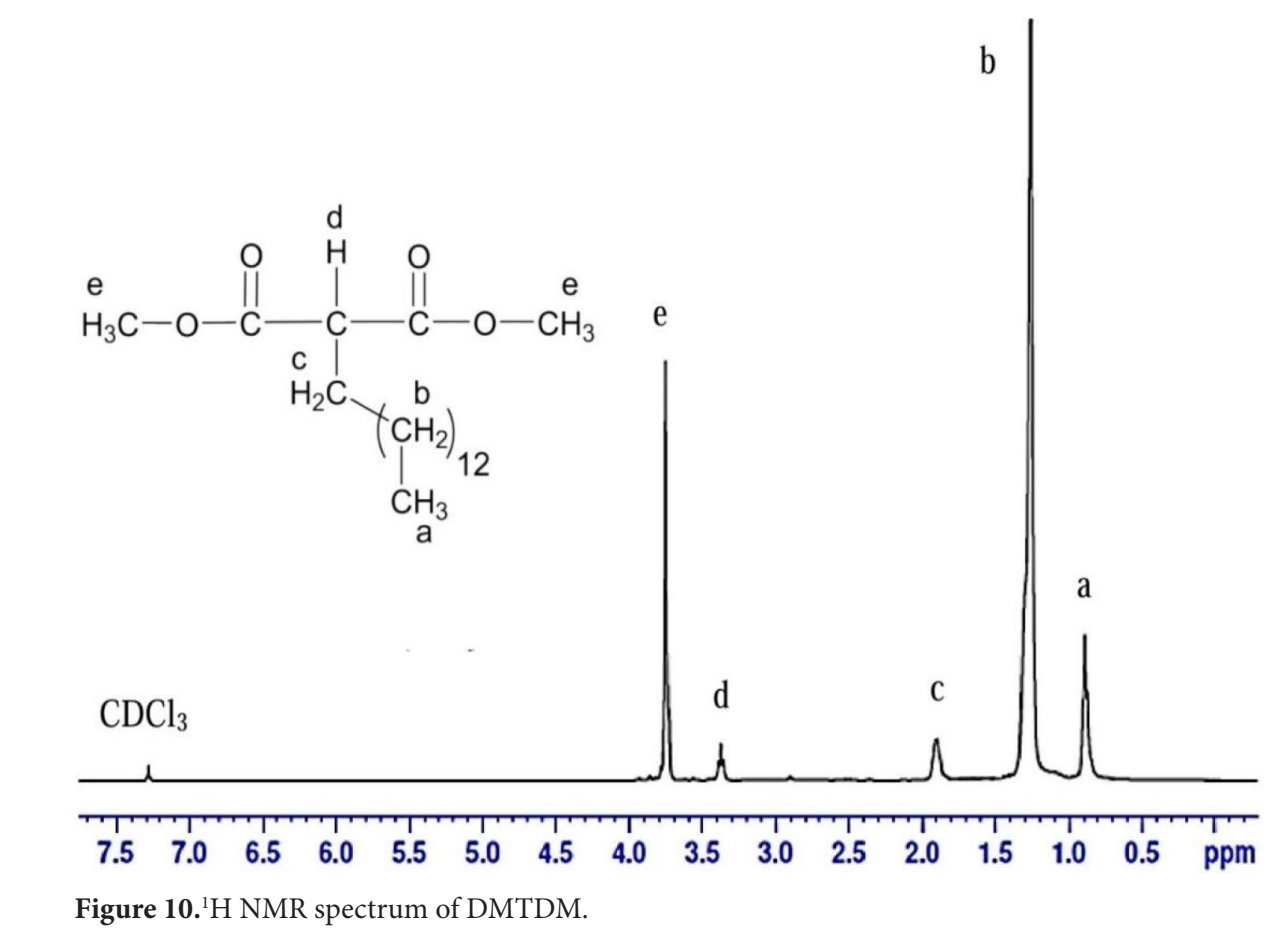
1H NMR spectrum of DMTDM.

The ^1^H NMR spectroscopy displays the major signal assignments of MP and DMTDM, as presented in Figure 10. The signal at 0.87–0.90 ppm could be assigned to the terminal methylene group (-C *H*_3_) of MP, which also appeared in DMTDM at 0.87–0.89 ppm next to the signal of terminal methyl (–CH_2_) at 1.26 ppm for both MP and DMTDM. However, β- and α-methyl carbon of the acidic group was deshielded and absorb downfield at about 1.61–1.63 ppm, 2.29–2.33 ppm for MP and 1.90–1.91 ppm, 3.35–3.39 ppm of DMTDM, respectively. The signal at 3.68 ppm and 3.75 ppm for MP and DMTDM, respectively, can be attributed to methyl ester (-COO-C *H*_3_) [9].

**Figure 11:**
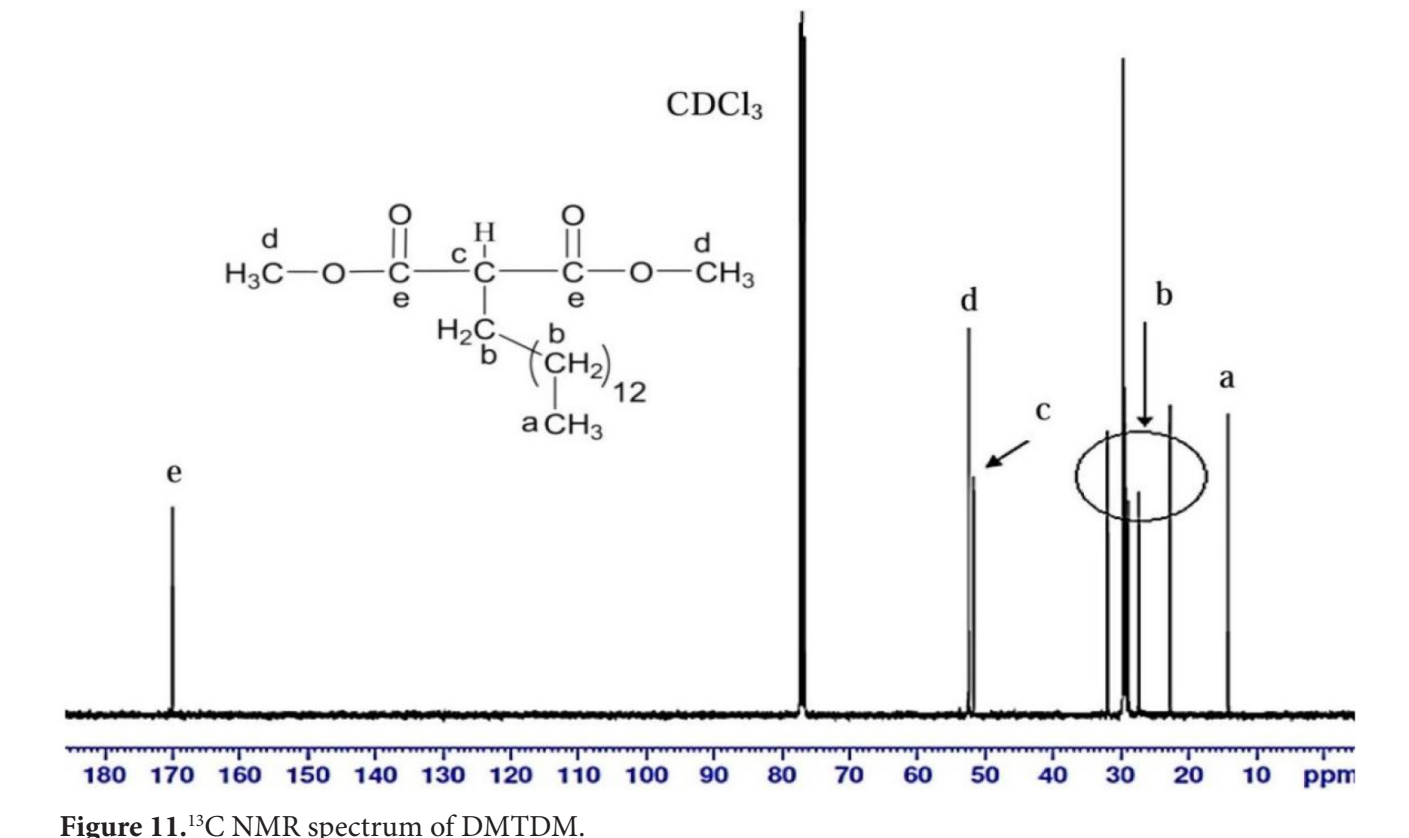
13C NMR spectrum of DMTDM.

Figure 11 show the ^13^C NMR spectrum of DMTDM. The signals at 170.04 ppm and 174.38 ppm denote the carbon atom of the carbonyl group (ester) of MP and DMTDM. The signals at 14.15–31.94 ppm refer to aliphatic carbon. However, the carbon atom attached to ester group was deshielded and absorb downfield at about 51.73 ppm and 52.40 ppm (CH_3_OOC- *C* HRCOOCH_3_). This result is in conformity with that reported by Kolb and Meier (2012) [9].

### 3.3. Synthesis of polyesters

**Figure 12:**
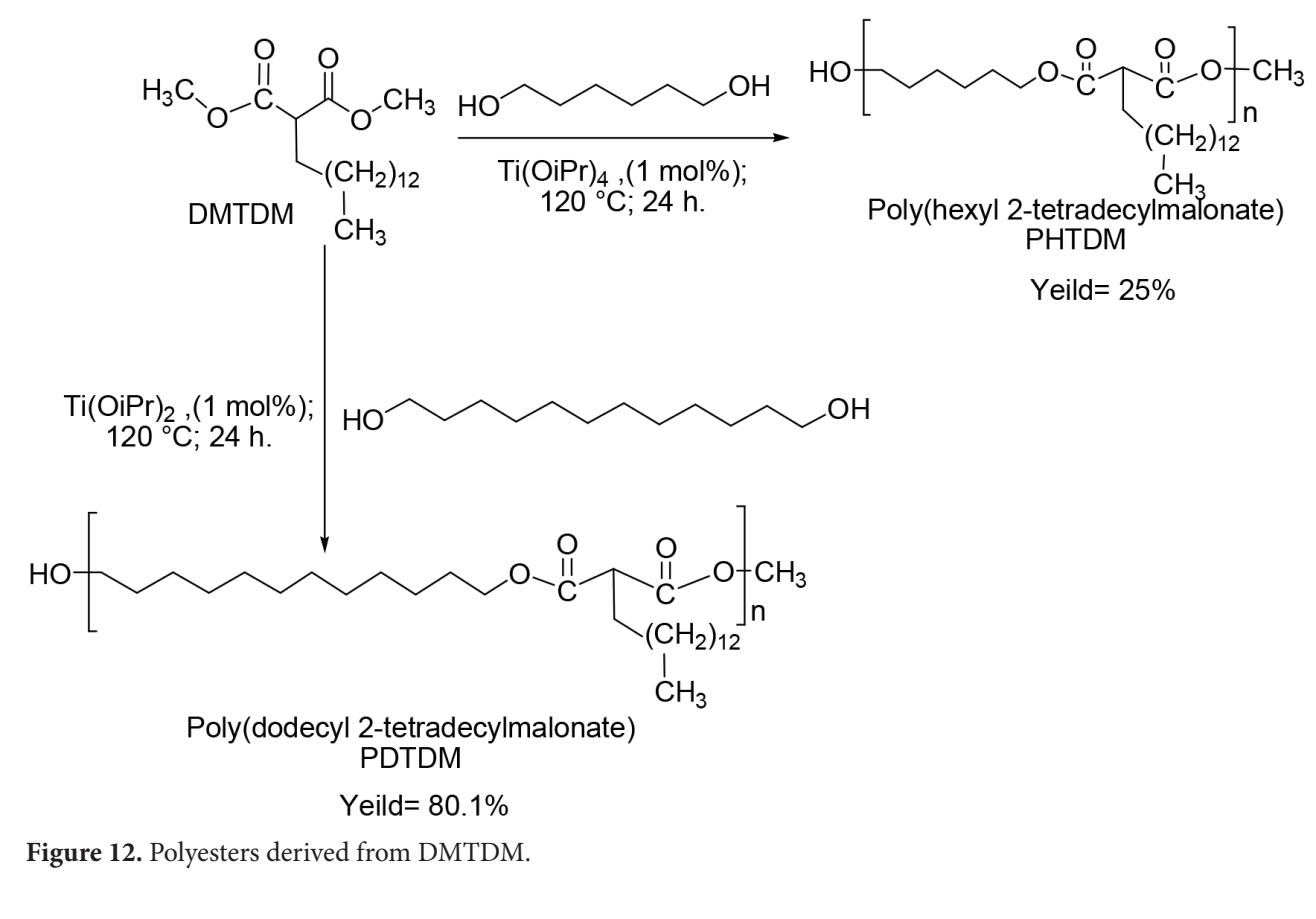
Polyesters derived from DMTDM.

After the malonate synthesis from saturated MP at optimal conditions, the monomer was polymerized to polyesters using different alcohols (Figure 12). Afterward, the polymerization performance of the corresponding polymers and their thermal behavior were investigated.

**Figure 13:**
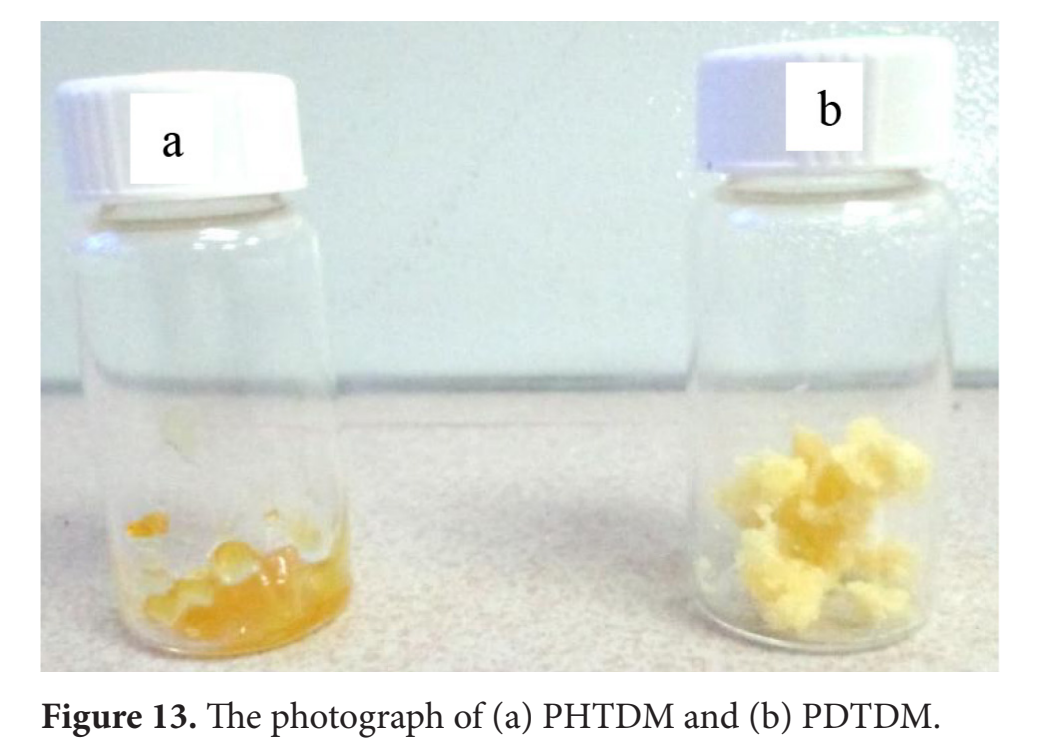
The photograph of (a) PHTDM and (b) PDTDM.

The obtained polyesters were dissolved in THF and then precipitated from methanol. As shown in Figure 13, PHTDM was yellowish, highly viscous and sticky at room temperature whereas PDTDM was solid at room temperature (but still very sticky and rubbery) due to the increasing chain length of diol from 6 carbon atoms to 12 carbon atoms. 

#### 3.3.1. FTIR analysis of PHTDM and PDTDM

**Figure 14:**
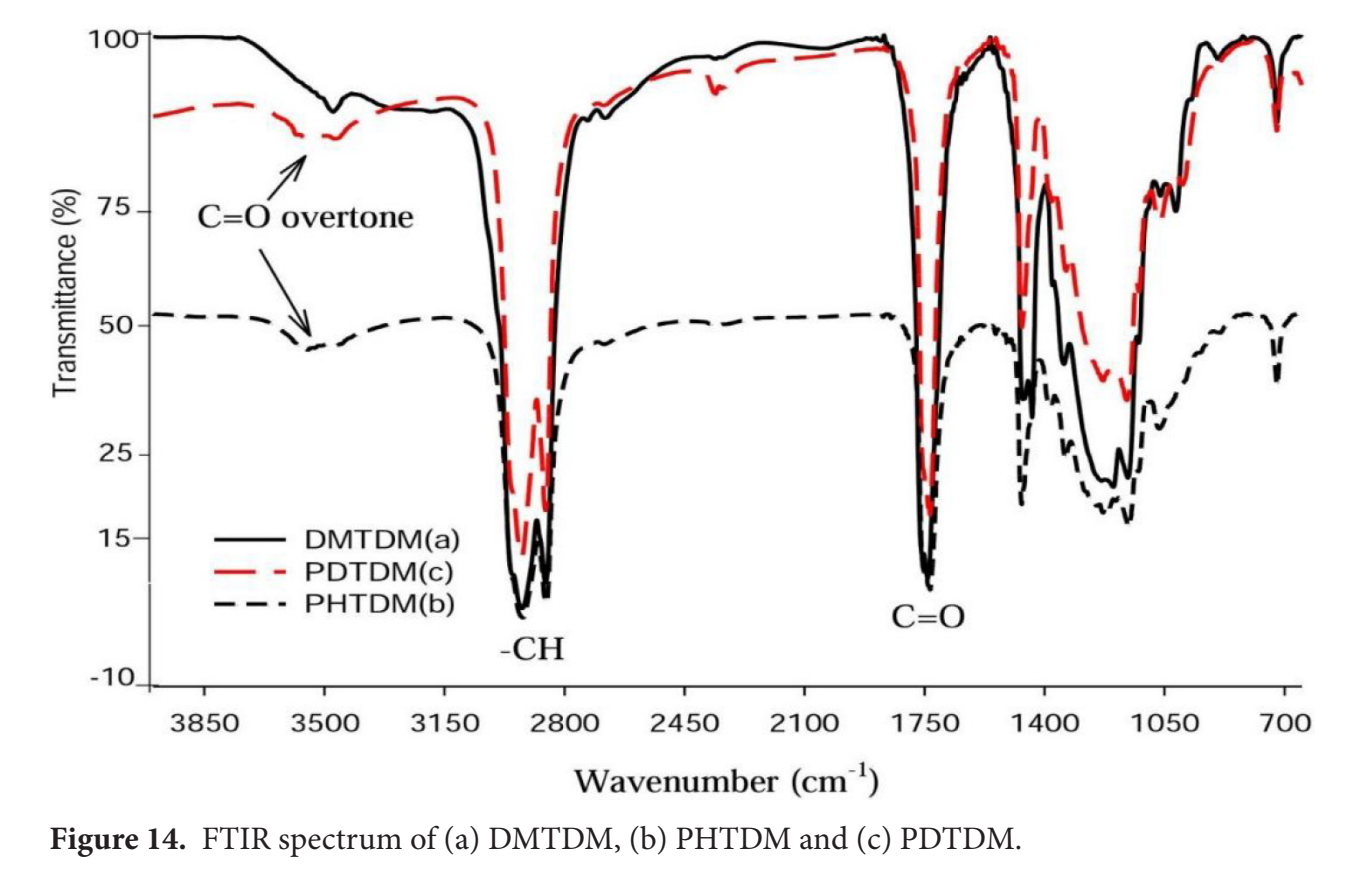
FTIR spectrum of (a) DMTDM, (b) PHTDM and (c) PDTDM.

Toverify the presence of carbonyl ester group in PHTDM and PDTDM, the final products were analyzed by FTIR. The FTIR spectrum of DMTHM, PHTDM and PDTDM is shown in Figure 14. The sharp absorption bands at 1740, 1733 and 1733 cm^–1^, for DMTHM, PHTDM and PDTDM, respectively, are assigned to functional groups of esterified carbonyl C=O. The peaks at 2922–2856 cm^–1^ indicate CH_2_ and CH_3_ stretching vibration of DMTHM, PHTDM and PDTDM. The absorption bands at 722 cm^–1^ signify C-H group vibration of DMTHM, PHTDM, and PDTDM. 

#### 3.3.2. NMR analysis of PHTDM and PDTDM 

**Figure 15:**
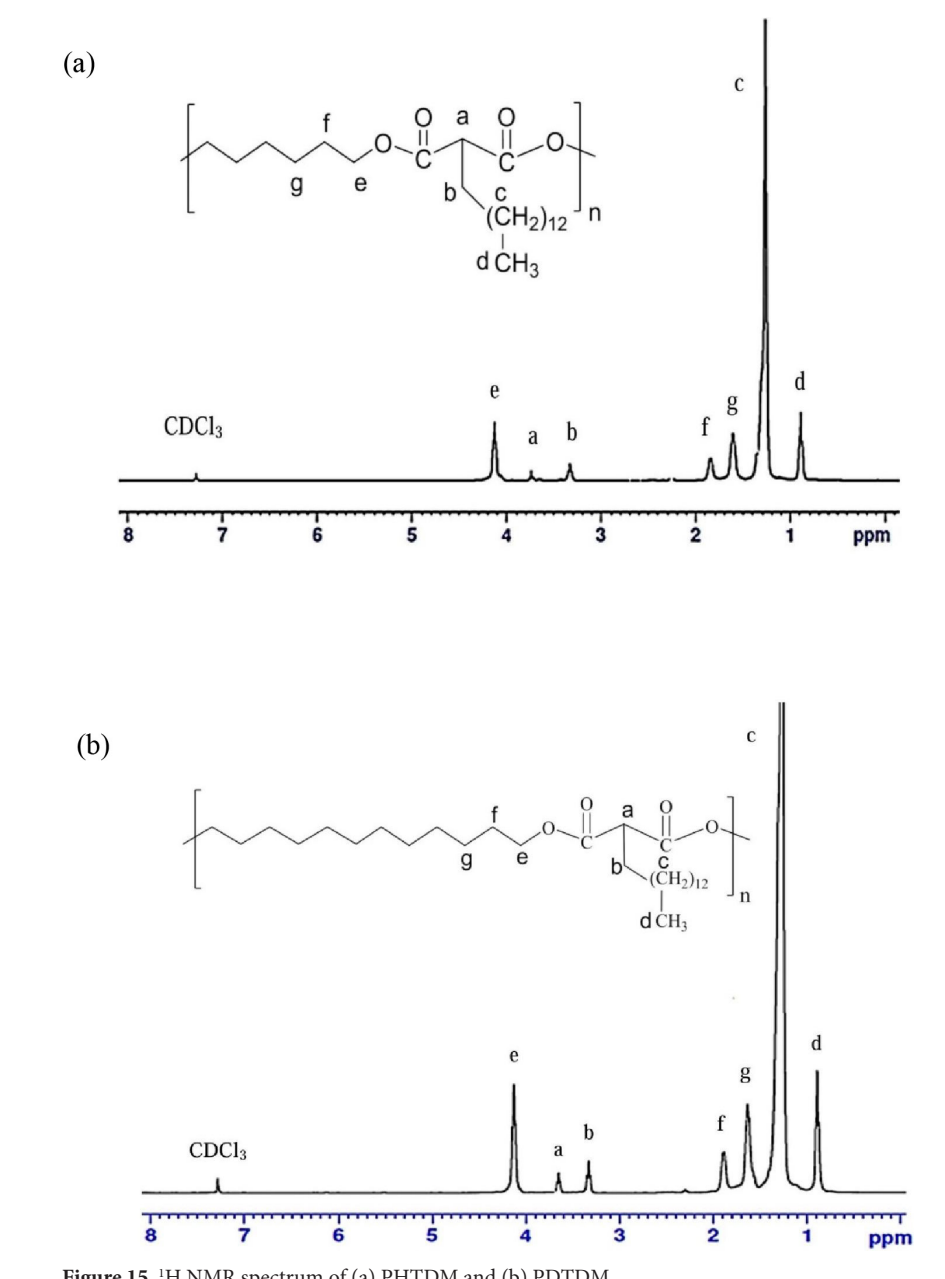
1H NMR spectrum of (a) PHTDM and (b) PDTDM.

The ^1^H NMR spectroscopy displays the main signal assignments of PHTDM and PDTDM as shown in Figure 15. The signal at 0.87–0.89 ppm could be assigned to the terminal methylene group (- *C* H *_3_* ) of PHTDM and PDTDM at 0.87–0.89 ppm next to the terminal methyl (–CH_2_) at 1.26 ppm which also appeared in of DMTDM. However, β-methyl carbon of the acidic group was deshielded and absorb downfield at about 1.90–1.91 ppm, 1.63 ppm, and 1.65 ppm for DMTDM, PHTDM, and PDTDM, respectively. The signal at 3.75 ppm can be attributed to methyl ester (-COO-CH_3_), which appears in DMTDM but is absent in PHTDM and DMTDM due to the polymerization. The spectra features observed for DMTDM, PHTDM and PDTDM are consistent with the study by Kolb and Meier (2012) [9].

**Figure 16:**
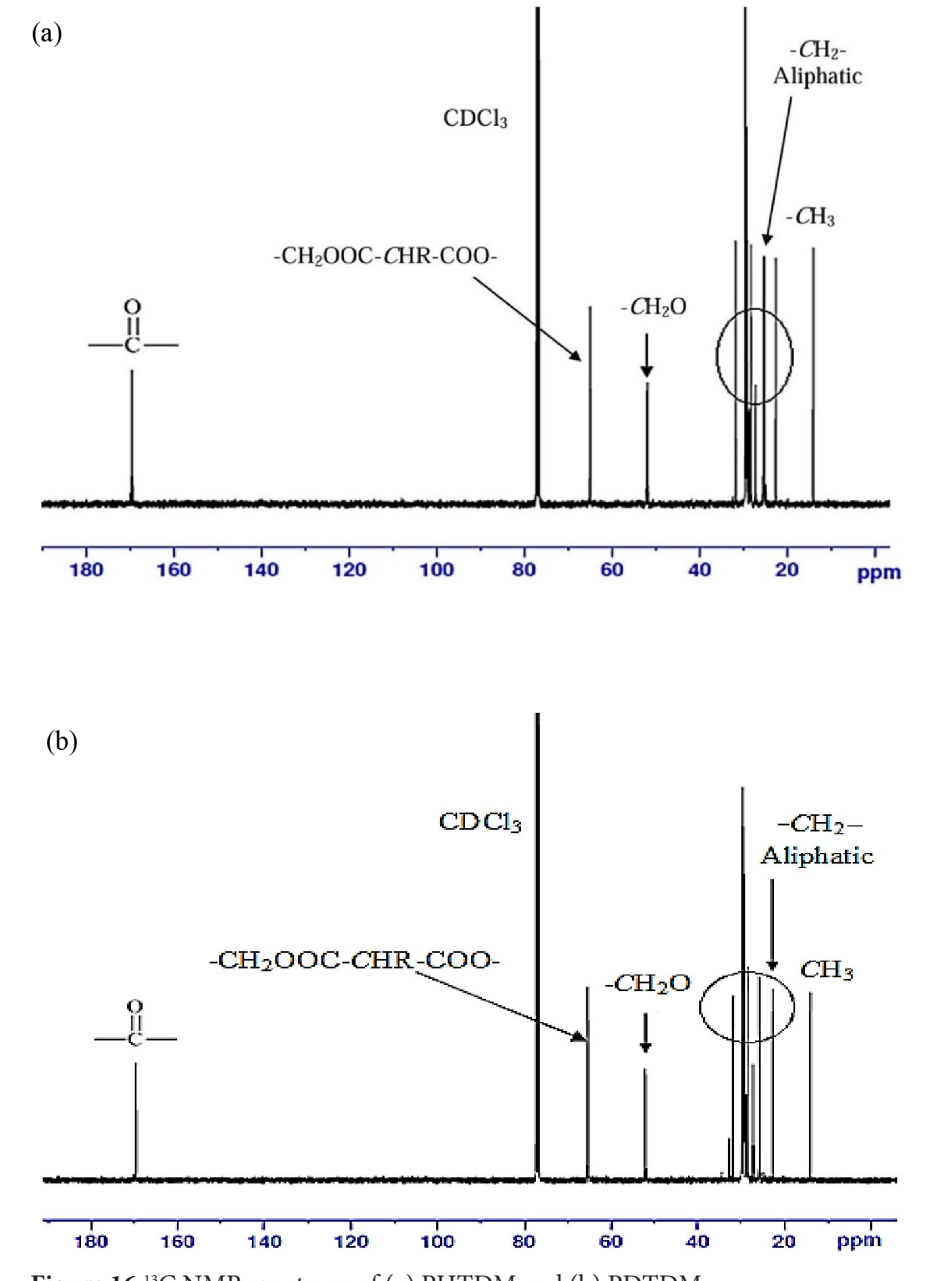
13C NMR spectrum of (a) PHTDM and (b) PDTDM.

Figure 16 shows the ^13^C NMR spectrum of PHTDM and PDTDM. The signals at 169.62 and 169.70 ppm indicate carbon atom of the carbonyl group (polyester) in PHTDM and PDTDM, respectively. The signals at 14.15–31.94, 14.13–31.93 and 14.14–32.01 ppm denote aliphatic carbon. However, the carbon atom attached to ester group deshielded and absorb downfield at 52.05 and 52.12 ppm (-CH_2_OOC-) for PHTDM and PDTDM, respectively. Furthermore, the atom carbon that attached to ester group was deshielded and absorb downfield at about 65.14 and 65.40 ppm (CH_3_OOC-CHRCOOCH_3_) in PHTDM and PDTDM, respectively.

#### 3.3.3. Thermal analysis of PHTDM and PDTDM

**Figure 17:**
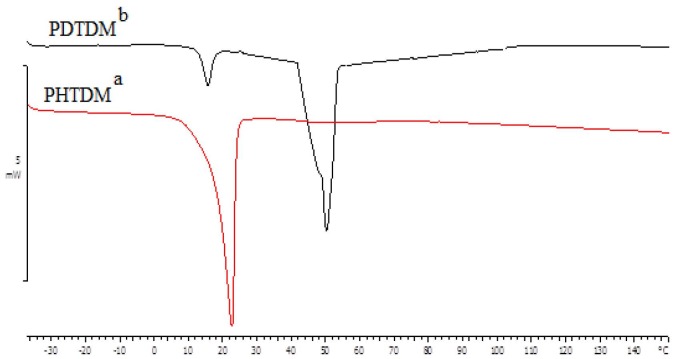
DSC thermogram of (a) PHTDM and (b) PDTDM.

The thermal behavior of the synthesized polyesters was investigated by DSC analysis in the range of temperature from –40 to 200 ºC (Table and Figure 17). As expected, PHTDM did not show any glass transition within that range but exhibited melting transition at 22 ºC. However, PDTDM showed glass transition at 13 ºC and melting transition at 51 ºC. It can be inferred from the DSC analysis that the side-chain length and the resulting nonpolar interactions are the major driving force for the crystallinity of the produced polyesters [9]. The obtained polyesters were well soluble in numerous common organic solvents such as acetone, tetrahydrofuran, chloroform, and toluene. 

**Table:** Thermal behavior and molecular weight distribution of PHTDM and PDTDM.

PHTDM a	PDTDM b	Properties
25%	80.1%	Yield (%)
-	13	Tg (°C)
13	51	Tm (°C)
5229	8919	–Mn (Da)
8096	12508	–Mw (Da)
1.5	1.4	PDI (Mw/Mn)

(a) Poly(hexyl 2-tetradecylmalonte); (b) poly(dodecyl 2-tetradecylmalonte); Tg = Glass transition temperature; Tm = Melting temperature; –Mn = Number-average molecular weight ; –Mw = Weight-average molecular weight.

## 4. Conclusion

In this study, bioplastics were synthesized from palm palmitic acid via the copolymerization of malonate derivative (DMTDM) with diol to yield poly(hexyl 2-tetradecylmalonte) (PHTDM) and poly(dodecyl 2-tetradecylmalo-nate) (PDTDM). The results showed that methyl palmitate could be easily converted to its respective malonate derivative using NaH as a base and in DMC as a reactive solvent. The malonate derivative was then easily copolymerized with 1,6-hexanediol or 1,12-dodecandiol to yield poly(hexyl 2-tetradecylmalonte) (PHTDM) or poly(dodecyl 2-tetradecylmalonte) (PDTDM), respectively. The thermal behavior and molecular weight of these polymers were investigated. Poly(hexyl 2-tetradecylmalonte) showed a poor yield due to its high solubility in common organic solvents, while PDTDM showed good thermal properties and high yield. Thus, it canbe inferred that the aliphatic side-chain of the methyl palmitate-derived malonate had a significant impact on the thermal properties due to its role as an internal plasticizer. This synthesis is an early stage in the synthesis of palm palmitic acid based polyesters, further properties will be investigated in future studies. In general, these synthetic polyesters can be used as internal plasticizers in bio-based industry.

## References

[ref1] (2015). Study on the spectrophotometric detection of free fatty acids in palm oil utilizing enzymatic reactions. Molecules.

[ref2] (2015). The challenges and prospects of palm oil based biodiesel in Malaysia. Energy.

[ref3] (2013). Recent advances in the production, recovery and applications of polyhydroxyalkanoates. Journal of Polymers and Environment.

[ref4] (2011). Design and analysis of poly-3-hydroxybutyrate production processes from crude glycerol. Process Biochemistry.

[ref5] (2020). Two-stage fermentation optimization for poly-3-hydroxybutyrate production from methanol by a new Methylobacterium isolate from oil fields. Journal of Applied Microbiology.

[ref6] (2011). Synthesis of polyhydroxyalkanoate from palm oil and some new applications. Applied Microbiology and Biotechnology.

[ref7] (2009). Extraction and physicochemical properties of low free fatty acid crude palm oil. Food Chemistry.

[ref8] (2007). The Lipid Handbook.

[ref9] (2009). Application of transmission FT-IR spectroscopy for the trans fat determination in the industrially processed edible oils. Food Chemistry.

[ref10] (2012). Monomers and their polymers derived from saturated fatty acid methyl esters and dimethyl carbonate. Green Chemistry.

[ref11] (2006). High-resolution 1H nuclear magnetic resonance in the study of oils. Modern Magnetic Resonance.

[ref12] (2015). Efficient biomass transformations catalyzed by graphene-like nanoporous carbons functionalized with strong acid ionic liquids and sulfonic groups. Green Chemistry.

[ref13] (2011). The acetoacetic ester condensation and certain related reactions. In: Organic reactions. Hoboken.

[ref14] (2018). The reactions of dimethyl carbonate and its derivatives. Green Chemistry.

[ref15] (2007). Carbanions and other carbon nucleophiles. Advanced Organic Chemistry.

[ref16] (2019). Counter-ion and solvent effects in the C- and O-alkylation of the phenoxide ion with allyl chloride. Journal of Physical Organic Chemistry.

